# Long-read DNA sequencing leads to the more complete sequence characterization of the fruit size reducing region flanking a Fusarium wilt resistance gene

**DOI:** 10.1186/s43897-022-00037-w

**Published:** 2022-07-02

**Authors:** Tong Geon Lee

**Affiliations:** 1 Horticultural Sciences Department, University of Florida, Gainesville, FL 32611 USA; 2 Gulf Coast Research and Education Center, University of Florida, Wimauma, Gainesville, FL 33598 USA; 3 Plant Breeders Working Group, University of Florida, Gainesville, FL 32611 USA; 4 Plant Molecular and Cellular Biology Graduate Program, University of Florida, Gainesville, FL 32611 USA

## Introduction

Fruit size is an important trait for fruit crops including tomato (*Solanum lycopersicum*). It influences yield, which is the top priority for plant breeding and improvement programs. Studies have shown that introgression of disease resistance, often a necessity for successful cultivar development, impacts negatively on yield (Ning et al., 2017). Therefore, genetic resources, which do not compromise existing traits except for the new trait of interest, are always in high demand as such resources can be highly beneficial for rapidly incorporating new trait(s) into breeding backgrounds. Given this, exploiting knowledge of these negative impacts at the DNA sequence level has been of interest in the (applied) plant science society.

To provide a rich sequence resource for the discovery of candidate(s) associated with fruit size reduction, we focus on the Fusarium wilt resistance *I-3* introgression (both the *I-3* gene and its flanking regions which typically cover multi-megabases), which has been incorporated from a wild tomato (*S. pennellii*; accession LA716) (Scott and Jones, 1989) into a domesticated tomato (*S. lycopersicum*) and is historically known to reduce fruit size (weight) of domesticated tomatoes (Scott 1999; Chitwood-Brown et al., 2021a). Interestingly, a recent, shortened *I-3* introgression obtained via crossing over(s) evidenced that the short introgression does not reduce fruit size, implying 1) the linkage drag constrained to reduce fruit size is broken and 2) gene(s) residing on the genomic region of wild tomato crossed over with the multi-megabases could be a primary cause of fruit size reduction. The identification of fruit size reduction-causing gene(s) is dependent on gene discovery over the genomic region, which has been crossed over and currently carries gaps with > 236-kbp ambiguous nucleotides based on the reference genome. Three tomatoes sharing genetic backgrounds except for the *I-3* introgression were chosen: resistant Fla. 8814 with the *I-3* introgression (estimated 4.2-Mbp), which shows reduced fruit size (hereafter, Fla. 8814^*Long*^), resistant Fla. 8814 with a different *I-3* introgression with a shorter interval (estimated 140-kbp) via crossing over(s), which does not show reduced fruit size (Fla. 8814^*Short*^), and susceptible Fla. 8814 with *i-3* allele, which also does not show reduced fruit size (Fla. 8814^*None*^).

Studies have provided evidence that genome assembly, especially via long reads, enhances the detection of sequence variants, importantly structural variants (SVs) (i.e., ≥50 bp in length) (Wang et al., 2021). Further, genetic variation between Fla. 8814 used in this study and Heinz 1706 used as a fully sequenced reference domesticated tomato (Tomato Genome Consortium, 2012) might lead to misinterpretation of variants and/or a failure to discover existing variants originally derived from a wild tomato if aligning fragments of sequence to the domesticated tomato genome is solely applied. We therefore sequenced the three tomato genomes using both Oxford Nanopore and Illumina NovaSeq technologies, and contigs were constructed on the basis of de novo assembly.

## Results

**Fig. 1:**
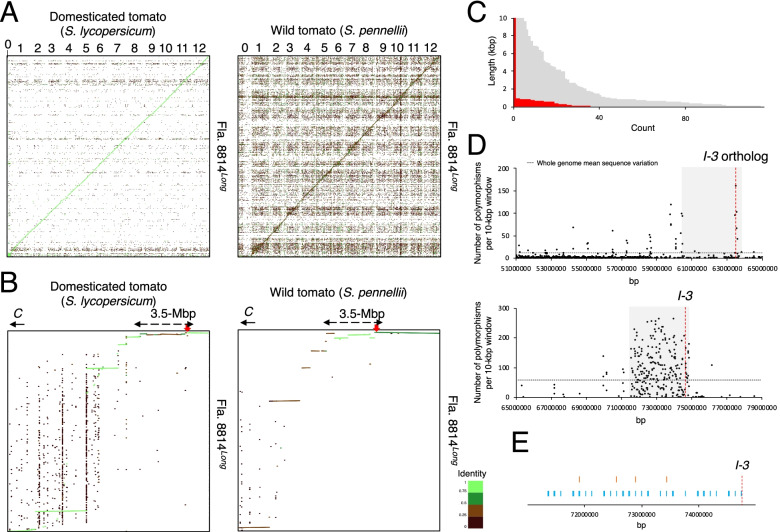
Sequence characterization of the Fusarium wilt resistance *I-3* flanking fruit size reducing region. **A** Dot plot comparison between the reference genome (horizontal) and contigs of Fla. 8814^*Long*^ (vertical) (**A** and **B**). Numbers represent the tomato chromosomes. Sequence similarity is color coded from 0 to 1 (**A** and **B**; The summary of identity is placed next to **B**). **B** Zoomed in plots (a 14-Mbp interval) capturing the *I-3* introgression on chromosome 7. Inferred *I-3* introgression (approximately 3.5-Mbp) is depicted by dash arrows. Red arrows indicate the position of the *I-3* gene. *C*: centromere. **C** Length distribution of 115 gaps found in a 4-Mbp interval of the wild tomato genome carrying the *I-3* introgression (gray color) and 36 gaps overlapped by contigs (red color). **D** Common sequence variant density plot of the Fusarium wilt resistance *I-3* introgression. Top and bottom panels show common sequence variants on the basis of alignment to a reference domesticated tomato (i.e., sequence variant found in both Fla. 8814^*Long*^ and Fla. 8814^*Short*^, but not in Fla. 8814^*None*^) and to a reference wild tomato (i.e., found in both Fla. 8814^*Short*^ and Fla. 8814^*None*^, but not in Fla. 8814^*Long*^), respectively. Inferred *I-3* introgression (approximately 3.5-Mbp) is depicted in gray. The approximate locations of *I-*3 ortholog (*Solyc07g055640* between 63,514,724 and 63,521,342 bp; top panel in **D**) and *I-3* (*Sopen07g029010* between 74,627,845 and 74,630,497 bp; bottom panel in **D** and **E**) are depicted by red lines. See Fig. S3 for the sequence variant density plot for each of Fla. 8814^*Long*^, Fla. 8814^*Short*^, and Fla. 8814^*None*^. Physical positions are based on individual reference genomes. **E** Locations of structural variants (SVs) (≥50 bp in length) found in Fla. 8814^*Long*^ only as compared with orthologous DNA sequences (i.e., found in neither Fla. 8814^*Short*^ nor Fla. 8814^*None*^) (orange vertical bars) and found in both Fla. 8814^*Short*^ and Fla. 8814^*None*^, but not in Fla. 8814^*Long*^ (blue vertical bars). Physical position is based on the reference genome of a wild tomato

We produced long-read genome sequence data of over 100 × genome coverage of each of three tomatoes (Table S1). De novo assembly coupled with short-read error correction gave the assembly of each tomato with contig N50 2.7 to 5.3-Mbp (Table S2). Based on the alignment of the short reads to the assembly, the high mapping rate (> 98%) and coverage rate (> 94%) indicated a high consistency between the assembly and the reads (Table S3). Further, each assembly had a BUSCO score at least 96.9% (Table S4), indicating high completeness of the assembly. Lastly, the alignment of assembled contigs to two reference genomes showed that there was a high degree of collinearity between the reference and the contigs at the macrolevel (Fig. 1A, Fig. S1).

Sequence alignment has placed several contigs in a 14-Mbp interval that carries to the *I-3* introgression (Fig. 1B, Fig. S2). Clearly, the centromere-proximal *I-3* flanking region shares less similarity with the domesticated tomato genome. In contrast, this centromere-proximal flanking region shares high similarity with that of the wild tomato genome. This observation is in agreement with a previous study reporting that the majority of remaining wild tomato sequences in domesticated tomato backgrounds are centromere-proximal. The current version of the wild tomato genome carries 115 gaps (i.e., ≥50 bp each) filled with ambiguous nucleotides (i.e., Ns) over an interval spanning 71 to 75-Mbp on chromosome 7. In the Fla. 8814^*Long*^, however, the alignment depicts that 36 of these gaps were overlapped by unambiguous sequences from large contigs (> 1.0-Mbp each) (Fig. 1C, Table. S5), thus making gap-free genome assembly more achievable.

High sequence variant frequency was apparent near the *I-3* from variant discovery based on alignment of contigs to reference genomes (Fig. 1D, Fig. S3, Table S6). By using data on the alignment of contigs from three tomatoes to the reference genome of a wild tomato as compared with a previous approach where sequence variants were indirectly inferred (Chitwood-Brown et al., 2021b), it is clear that the size of *I-3* introgression is close to 3.5-Mbp (between 60.4 and 63.7-Mbp, and between 71.4 and 74.6-Mbp in the domesticated and wild tomatoes, respectively). Simultaneously, 72 SVs were uniquely identified within a 3.5-Mbp *I-3* flanking interval of Fla. 8814^*Long*^ compared with the same sized intervals of Fla. 8814^*Short*^ and Fla. 8814^*None*^ sharing similarity with the 3.5-Mbp of Fla. 8814^*Long*^ (Fig. 1E, Table S7). Interestingly, a SV (starting at position 72,195,816 bp on chromosome 7 of wild tomato) found in both Fla. 8814^*Short*^ and Fla. 8814^*None*^, but not in Fla. 8814^*Long*^, encompasses part of the exonic region of a wild tomato gene *Sopen07g026470* (showing similarity to a kruppel-like factor *Solyc07g052913*). For a shortened *I-3* introgression, an interval with the continuous high sequence variants (approximate 150-kbp between 63.43 and 63.58-Mbp in the domesticated tomato) was observed, similar to what has been estimated previously (Table S8).

## Discussion

In the current study, we report two major contributions. First, many of the missing DNA sequences identified in *I-3* flanking regions have been sequence-resolved by assembling long-read data. The current reference genome of LA716 was assembled using the Illumina short paired-end/mate-pair and BAC-end sequencing (Bolger et al., 2014). Our contigs together with SVs identified within the wild tomato introgression now provide access to previously unidentified regions of tomato genetic variation. Second, the *I-3* introgression in Fla. 8814^*Long*^ is most likely to be close to 3.5-Mbp. Determination of accurate introgression boundaries is challenging with current genomic technologies. It is further hindered by another existing wild tomato introgression(s) (left panel in Fig. S3). Given the calculated recombination rates (1.0 to 2.6 cM/Mbp) in this U.S. large-fruited (round) fresh-market tomato class and the lower level of recombination between domesticated and wild tomato species is generally observed than that of a cross between two domesticated tomatoes (Bhandari and Lee, 2021; Bhandari et al., 2022), limited crossing over points may exist near the *I-3* locus.

A continuous stretch of gaps across the *I-3* flanking regions indicates complex regions of genetic variation near this disease resistance locus. Other long-read and ultra-long-read sequencing platforms coupled with fully sequenced large-insert clones such as bacterial artificial chromosome/Fosmid can be required in order to identify the complete spectrum of genetic variation near the *I-3*, which reduces fruit size in this model fruit crop. Advances in assembly and bioinformatics technologies may also uncover previously unassembled genomic sequences and correct erroneous sequences.

### Supplementary Information

**Additional file 1.** Materials and Methods.

**Additional file 2: Fig. S1.** Dot plot comparison between the reference genome (horizontal) and contigs of Fla. 8814^*Short*^ and Fla. 8814^*None*^ (vertical). Top and bottom plots use the domesticated and wild tomato genomes as target sequences, respectively. Sequence similarity is color coded from 0 to 1.

**Additional file 3: Fig. S2.** Zoomed in plots (a 14-Mbp interval) capturing the *I-3* introgression on chromosome 7. Sequence similarity is color coded from 0 to 1. *C*: centromere.

**Additional file 4: Fig. S3.** Sequence variant density plot of the *I-3* introgression. Left and right panels show sequence variants on the basis of alignment to a reference domesticated tomato and to a reference wild tomato, respectively. High sequence variant frequency between 59.5 and 61.0-Mbp in Fla. 8814 (left panel) indicates another existing wild tomato introgression(s) (Chitwood-Brown et al., 2021b; S.F. Hutton, personal communication). Inferred *I-3* introgression (approximately 3.5-Mbp) is depicted in gray. In the Fla. 8814^*Short*^, sequence variant frequency peaked near 63.52-Mbp, where the *I-3* ortholog *Solyc07g05540* (63,514,724 to 63,521,342 bp) is located (left panel). Physical positions are based on individual reference genomes.

**Additional file 5: Table S1.** Statistics of sequence data.

**Additional file 6: Table S2.** Statistics of contigs.

**Additional file 7: Table S3.** Statistics of short-read mapping.

**Additional file 8: Table S4.** Benchmarking Universal Single-Copy Orthologs (BUSCO) assessment.

**Additional file 9: Table S5.** Gaps found in a 4-Mbp interval spanning 71 to 75-Mbp on chromosome 7 of wild tomato.

**Additional file 10: Table S6.** Sequence variant frequency in a 14-Mbp interval on the basis of alignment to a reference domesticated tomato.

**Additional file 11: Table S7.** Structural variants identified within a 3.5-Mbp *I-3* flanking interval of Fla. 8814^*Long*^ compared with Fla. 8814^*Short*^ and Fla. 8814^*None*^

**Additional file 12: Table S8.** Genes residing on a shortened *I-3* introgression.

## Data Availability

The genome assemblies have been deposited in GenBank with the accession code PRJNA841967.
