# Supplementary material for: Long-read DNA sequencing leads to the more complete sequence characterization of the fruit size reducing region flanking a Fusarium wilt resistance gene
Source: Mol Hortic. 2022 Jul 2;2:16. doi: 10.1186/s43897-022-00037-w (PMC10514935; doi:10.1186/s43897-022-00037-w)
Supplement: Supplementary file 1 — Additional file 1. Materials and Methods. [file 43897_2022_37_MOESM1_ESM.docx]

**Long-read DNA sequencing leads to the more complete sequence characterization of the fruit size reducing region flanking a Fusarium wilt resistance gene**

Tong Geon Lee

**Materials and Methods**

All tomato material (Fla. 8814*^Long^*, Fla. 8814*^Shor^*^t^, and Fla. 8814*^None^*) was registered and stored by the University of Florida (UF) Institute of Food and Agricultural Sciences (IFAS) tomato breeding program (UF/IFAS, 2022). A single plant of each tomato was used for the DNA extraction. Genomic DNA extraction was performed as described in our previous study (Cook et al., 2012). The Nanopore (Ligation Sequencing Kit SQK-LSK110) and Illumina (DNA fragment length was 350 bp) libraries were prepared from the extracted DNA, and sequenced using the PromethION (R9.4.1 flow cell) and Illumina NovaSeq (2 × 150 bp paired-end) technologies, respectively (Novogene, Beijing, China).

The quality of the raw reads was controlled using NextCorrect (<https://github.com/Nextomics/NextDenovo>) and FastQC (www.bioinformatics.babraham.ac.uk/projects/fastqc) for the Nanopore and Illumina sequencing platforms, respectively. The Nanopore reads were assembled using NextDenovo (version 2.4; <https://github.com/Nextomics/NextDenovo>). Potential error in the resultant assembly was corrected using NextPolish (version 1.5; Hu et al., 2019) with the aid of the Illumina reads aligned using BWA (version 0.7.8; Li and Durbin, 2010). To assess the completeness of assembly, Benchmarking Universal Single-Copy Orthologs (BUSCO) (version 4.1.2; Simão et al., 2015) was used. In addition, BWA was used to map reads to each of assembled genome to assess the assembly quality.

minimap2 (version 2.24; Li, 2018) was used to align the final assembly of each sample to two tomato reference genomes, one from a domesticated tomato (*S. lycopersicum* cultivar Heinz 1706) (version SL4.0; Fernandez-Pozo et al., 2015) and the other from a wild tomato (*S. pennillii*; accession LA716) (data accessed 16 March 2022; Bolger et al., 2014). Candidate contigs mapping to the *I-3* flanking regions were further evaluated using progressiveMauve (version 2.3.1; Darling et al., 2010) and BLAST (i.e., blastn to the local database; Altschul et al., 1990). Comparison of identified contigs was visualized using D-GENIES (version 1.3.0; Cabanettes and Klopp, 2018). Detection of sequence variation (single-nucleotide variants, insertions-deletions, and SVs) was performed using SAMtools (version 1.9; Li et al., 2009). We limited our analysis to gaps with at least 1-kbp of flanking region matching the Fla. 8814*^Long^* contig. To infer the introgression boundaries, sequence variation frequency was calculated as previously described (Chitwood-Brown et al., 2021b).

**References**

Altschul SF, Gish W, Miller W, Myers EW, Lipman DJ. Basic local alignment search tool. J Mol Biol. 1990;215:403-10. doi:10.1016/S0022-2836(05)80360-2.

Cabanettes F, Klopp C. D-GENIES: dot plot large genomes in an interactive, efficient and simple way. PeerJ. 2018;6:e4958. doi:10.7717/peerj.4958.

Cook DE, Lee TG, Guo X, Melito S, Wang K, Bayless AM, Wang J, Hughes TJ, Willis DK, Clemente TE, Diers BW, Jiang J, Hudson ME, Bent AF. Copy number variation of multiple genes at *Rhg1* mediates nematode resistance in soybean. Science. 2012;338:1206-9. doi:10.1126/science.1228746.

Darling AE, Mau B, Perna NT. progressiveMauve: multiple genome alignment with gene gain, loss and rearrangement. PLoS One. 2010;5:e11147. doi:10.1371/journal.pone.0011147.

Fernandez-Pozo N, Menda N, Edwards JD, Saha S, Tecle IY, Strickler SR, Bombarely A, Fisher-York T, Pujar A, Foerster H, Yan A, Mueller LA. The Sol Genomics Network (SGN)--from genotype to phenotype to breeding. Nucleic Acids Res. 2015;43(Database issue):D1036-41. doi:10.1093/nar/gku1195.

Hu J, Fan J, Sun Z, Liu S. NextPolish: a fast and efficient genome polishing tool for long-read assembly, Bioinformatics. 2020,36: 2253–5.

Li H, Handsaker B, Wysoker A, Fennell T, Ruan J, Homer N, Marth G, Abecasis G, Durbin R; 1000 Genome Project Data Processing Subgroup. The Sequence Alignment/Map format and SAMtools. Bioinformatics. 2009;25:2078-9. doi:10.1093/bioinformatics/btp352.

Li H, Durbin R. Fast and accurate long-read alignment with Burrows-Wheeler transform. Bioinformatics. 2010;26:589-95. doi:10.1093/bioinformatics/btp698.

Li H. Minimap2: pairwise alignment for nucleotide sequences, Bioinformatics. 2018;34:3094–3100. doi:org/10.1093/bioinformatics/bty191.

Simão FA, Waterhouse RM, Ioannidis P, Kriventseva EV, Zdobnov EM. BUSCO: assessing genome assembly and annotation completeness with single-copy orthologs. Bioinformatics. 2015;31:3210-2. doi:10.1093/bioinformatics/btv351.

University of Florida, Institute of Food and Agricultural Sciences. UF/IFAS tomato breeding program. 6 April 2022. <https://tombreeding.ifas.ufl.edu>
