# Supplementary material for: Long-read DNA sequencing leads to the more complete sequence characterization of the fruit size reducing region flanking a Fusarium wilt resistance gene
Source: Mol Hortic. 2022 Jul 2;2:16. doi: 10.1186/s43897-022-00037-w (PMC10514935; doi:10.1186/s43897-022-00037-w)
Supplement: Supplementary file 2 — Additional file 2: Fig. S1. Dot plot comparison between the reference genome (horizontal) and contigs of Fla. 8814Short and Fla. 8814None (vertical). Top and bottom plots use the domesticated and wild tomato genomes as target sequences, respectively. Sequence similarity is color coded from 0 to 1. [file 43897_2022_37_MOESM2_ESM.pdf]

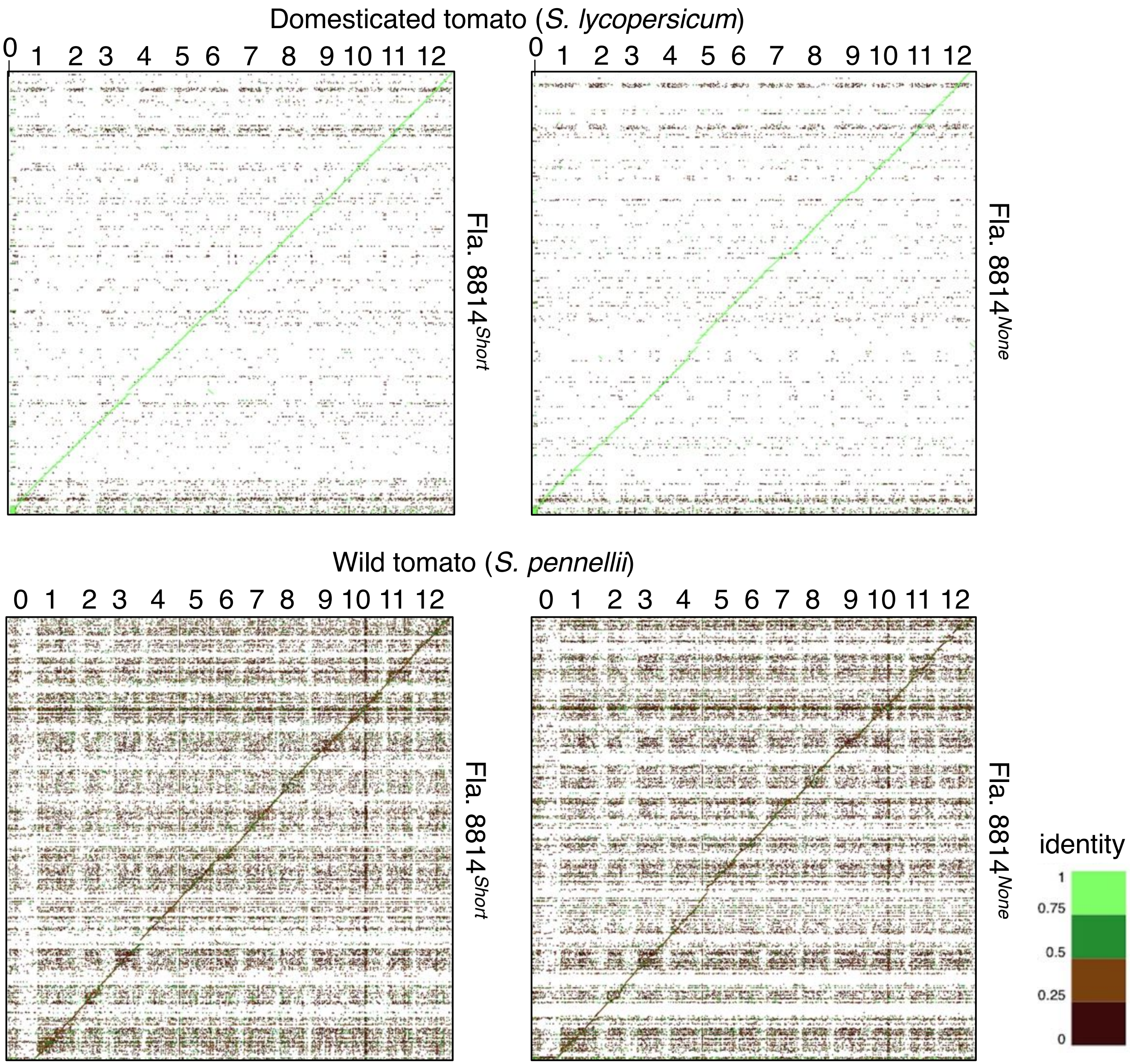

Domesticated tomato (*S. lycopersicum*)
0 1 2 3 4 5 6 7 8 9 10 11 12
Fla. 8814^Short
0 1 2 3 4 5 6 7 8 9 10 11 12
Fla. 8814^None
Wild tomato (*S. pennellii*)
0 1 2 3 4 5 6 7 8 9 10 11 12
Fla. 8814^Short
0 1 2 3 4 5 6 7 8 9 10 11 12
Fla. 8814^None
identity
1
0.75
0.5
0.25
0
