# Supplementary figures and images for: Long-read DNA sequencing leads to the more complete sequence characterization of the fruit size reducing region flanking a Fusarium wilt resistance gene
Source: Mol Hortic. 2022 Jul 2;2:16. doi: 10.1186/s43897-022-00037-w (PMC10514935; doi:10.1186/s43897-022-00037-w)

Domesticated tomato (*S. lycopersicum*)

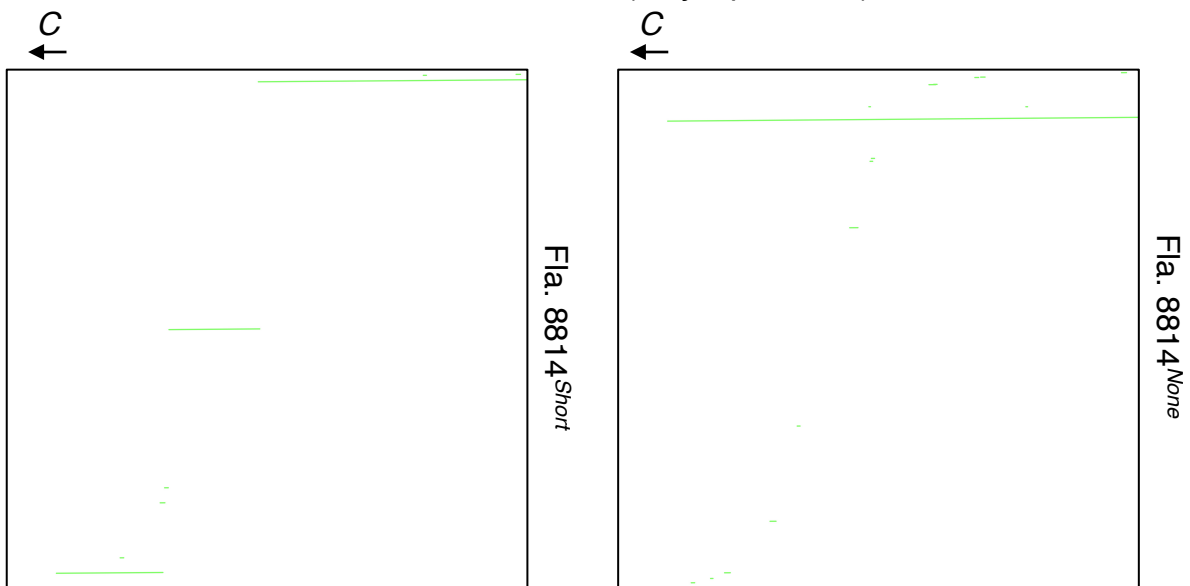

Wild tomato (*S. pennellii*)

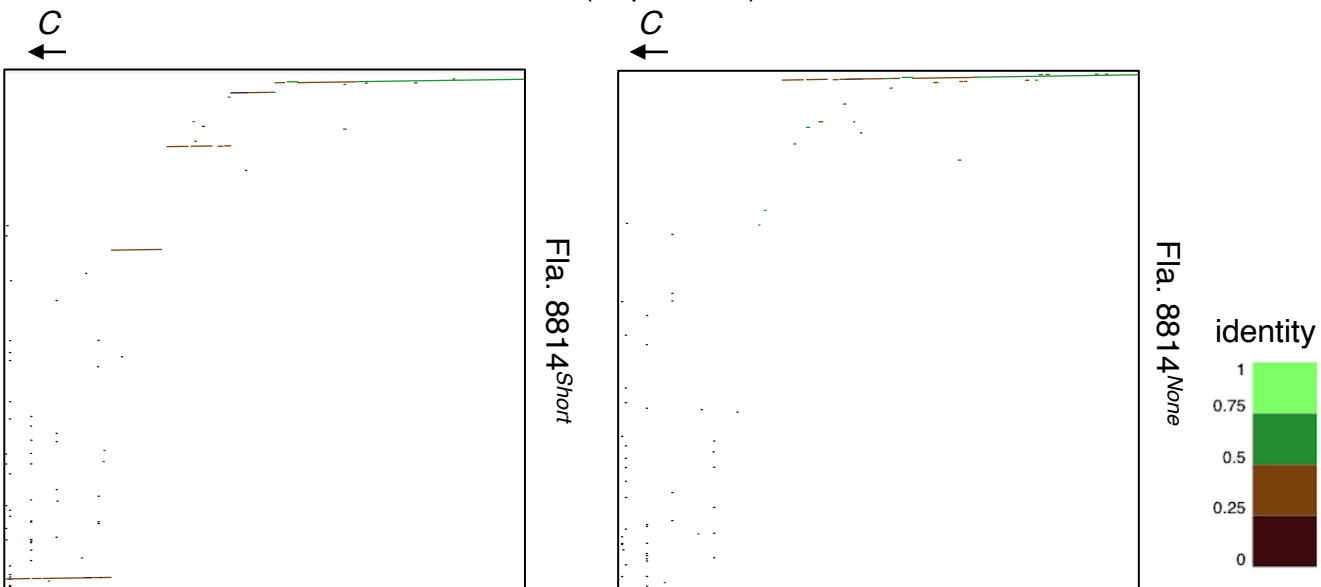

Supplement: Supplementary file 3 — Additional file 3: Fig. S2. Zoomed in plots (a 14-Mbp interval) capturing the I-3 introgression on chromosome 7. Sequence similarity is color coded from 0 to 1. C: centromere. [file 43897_2022_37_MOESM3_ESM.pdf]

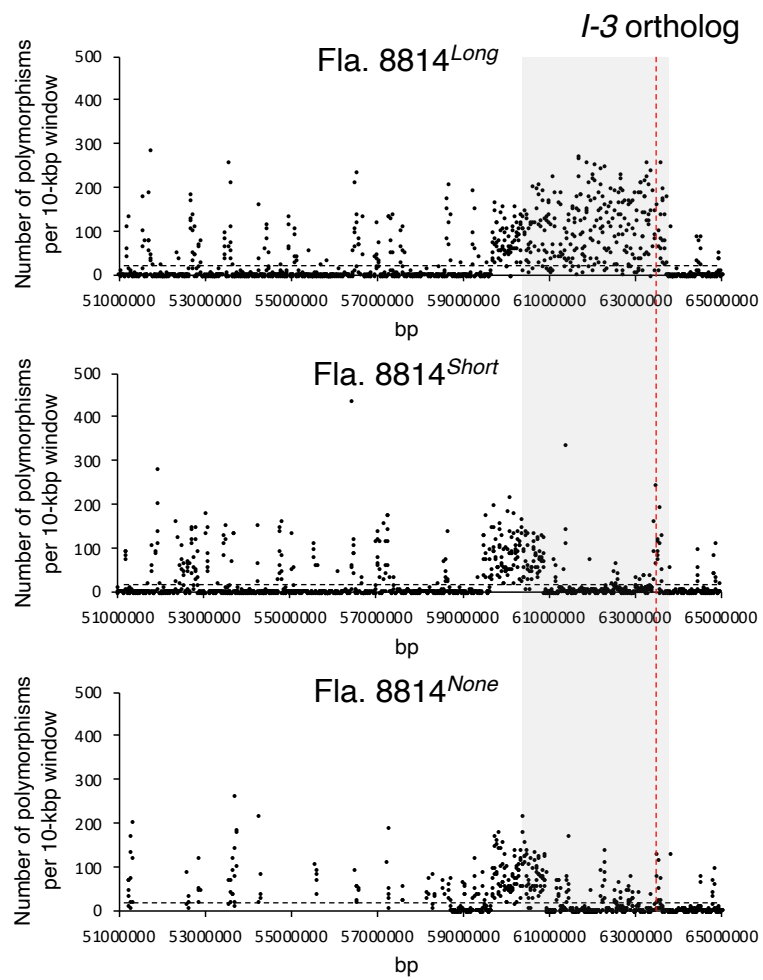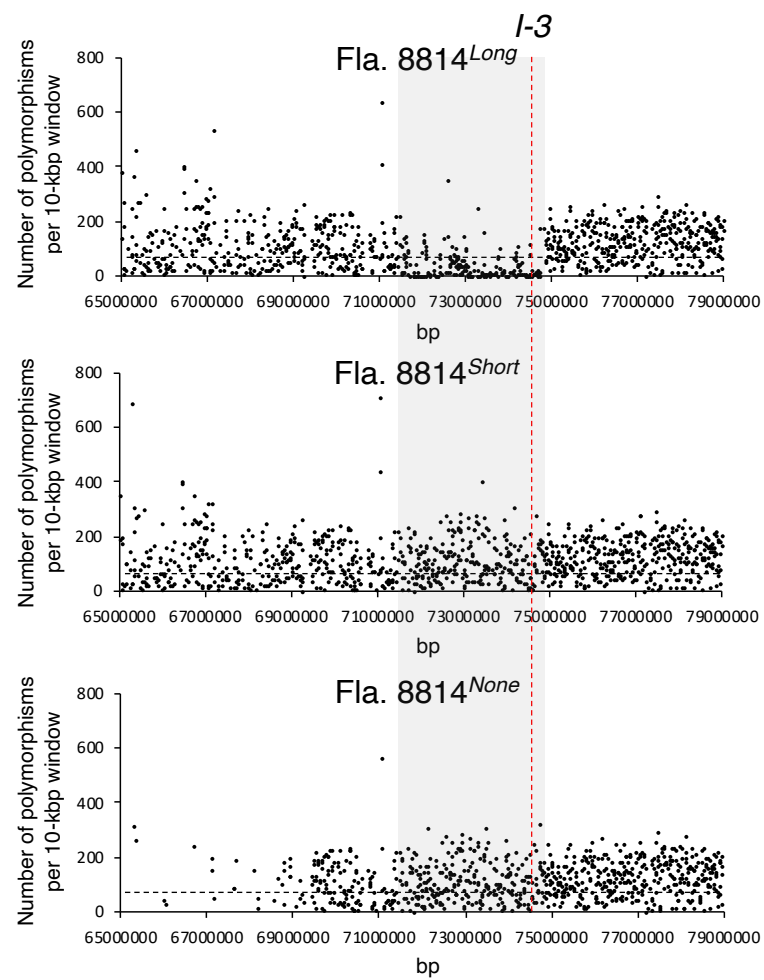

-- Whole genome mean sequence variation

Supplement: Supplementary file 4 — Additional file 4: Fig. S3. Sequence variant density plot of the I-3 introgression. Left and right panels show sequence variants on the basis of alignment to a reference domesticated tomato and to a reference wild tomato, respectively. High sequence variant frequency between 59.5 and 61.0-Mbp in Fla. 8814 (left panel) indicates another existing wild tomato introgression(s) (Chitwood-Brown et al., 2021b; S.F. Hutton, personal communication). Inferred I-3 introgression (approximately 3.5-Mbp) is depicted in gray. In the Fla. 8814Short, sequence variant frequency peaked near 63.52-Mbp, where the I-3 ortholog Solyc07g05540 (63,514,724 to 63,521,342 bp) is located (left panel). Physical positions are based on individual reference genomes. [file 43897_2022_37_MOESM4_ESM.pdf]
